# Novel use of Steinman pin in removal of broken interlocking screws

**DOI:** 10.1186/1757-1626-1-317

**Published:** 2008-11-17

**Authors:** Kiran Singisetti, George P Ashcroft

**Affiliations:** 1 Department of Orthopaedics, Woodend Hospital, Eday Road, Aberdeen, AB15 6XS, UK

## Abstract

Broken screws after interlocking nailing of long bones are commonly seen in Orthopaedic practice. Removal of such screws can be difficult particularly the distal part which is often held within the bone. We describe a simple technique of using Steinman pin to aid removal of broken screws in a case of non-union fracture tibia with broken interlocking nail and screws. Steinman pin being easily available and the reproducible technique make it a useful aid for removal of broken interlocking screws.

## Introduction

Broken interlocking screws are not an unusual problem in Orthopaedic practice and its causes can be varied [1,2]. While it is relatively easier to remove the head end of the screw with a screw-driver, it is difficult to remove the distal (tip end) part of the broken screw held within the bone. We describe a simple technique, with the use of Steinman pin to aid removal of such screws.

## Case presentation

A 28 year old male presented with increasing leg pain and disability after a previous interlocking nailing procedure for tibia shaft fracture. Radiographs of his leg showed the broken interlocking nail and screws in the tibia along with the non-union of fracture. To proceed with any revised fixation of the fracture required removal of the original metal work in situ, including the broken interlocking screws.

An appropriate incision was made over the screw and the head part of the broken screw removed after dissection. The blunt end of the Steinman pin was then passed down the screw track until it touched the broken end of retained screw 1. After checking the position using image intensifier the pin was struck with a mallet until the broken screw fragment is driven out of the bone 2. This part of the screw was then fished out from the soft tissues through a separate incision once it had been disimpacted from the nail and bone 3. Care should be taken to avoid damage of neurovascular structures while attempting removal of such screws.

## Conclusion

Several techniques and methods have been described for removal of broken interlocking nails and screws [2,3]. The simple and reproducible procedure of disimpacting the broken screws and easy availability of Steinman pin in operation theatres makes our technique practical to use.

## Consent

Written informed consent was obtained from the patient for publication of this case report and accompanying images. A copy of the written consent is available for review by the Editor-in-Chief of this journal.

## Competing interests

The authors declare that they have no competing interests.

**Figure 1:**
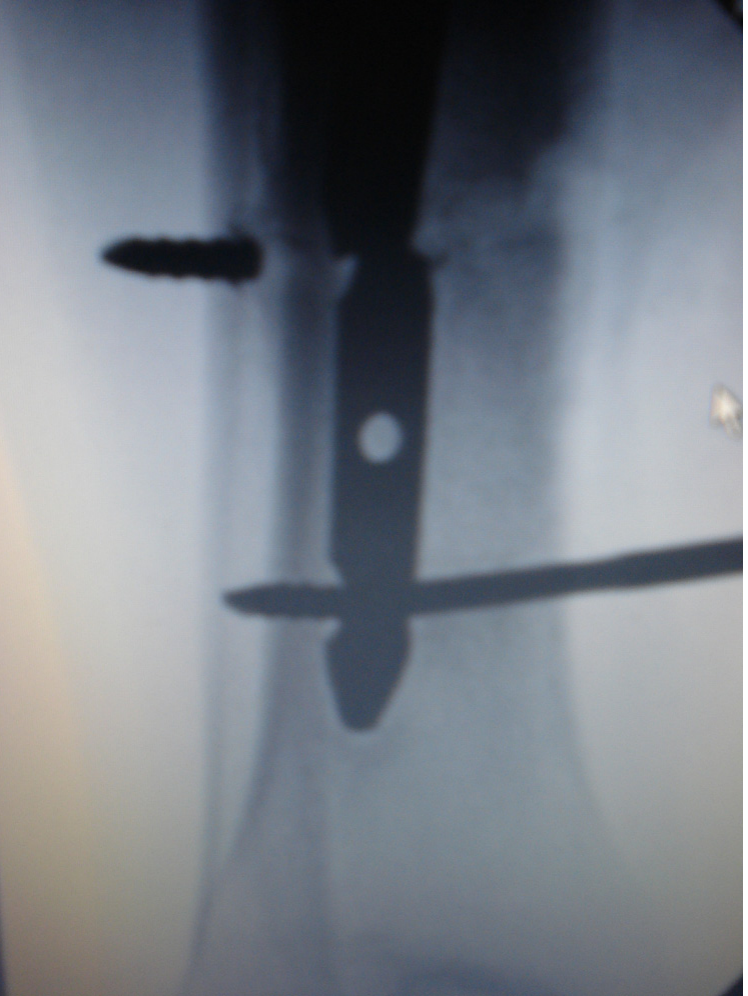
Image intensifier film to show broken screw with Steinman pin insertion.

**Figure 2:**
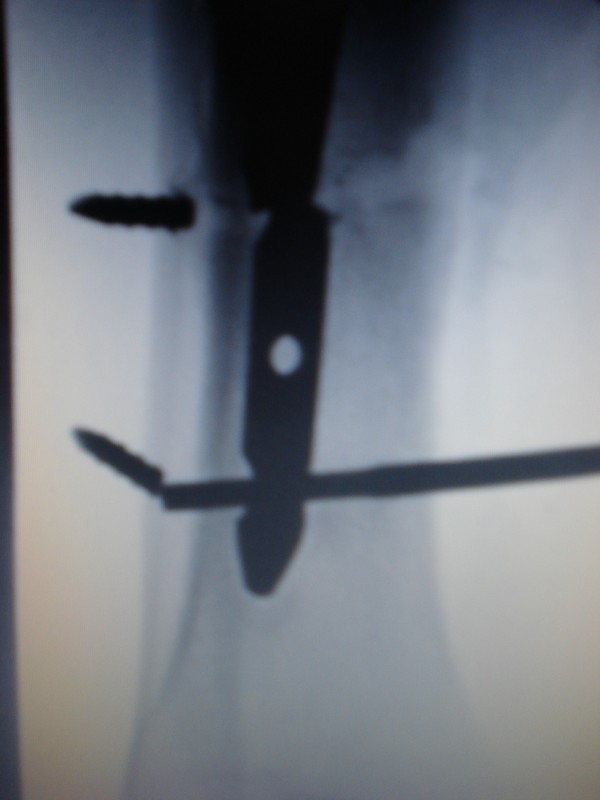
Image intensifier film to show disimpaction of broken screw from bone.

**Figure 3:**
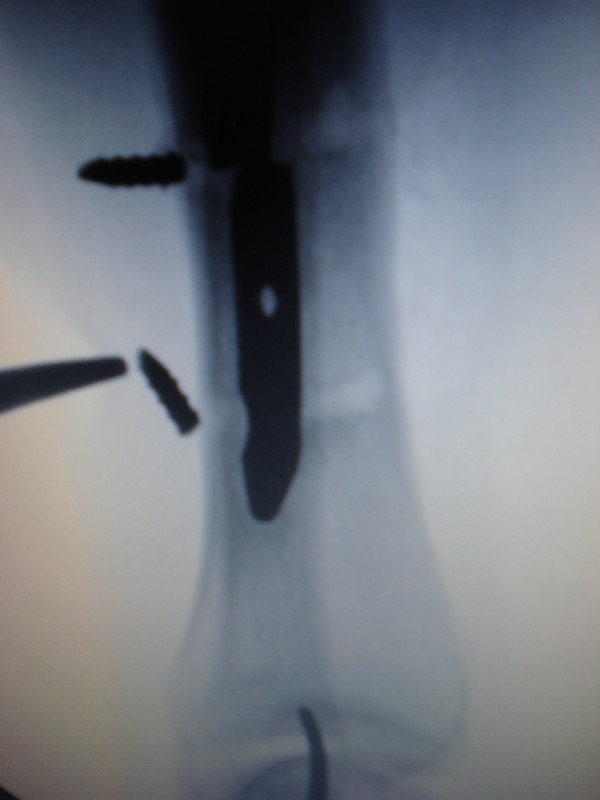
Image intensifier film to show removal of broken screw from soft tissues.

## Authors' contributions

KS was involved with the initial writing of the manuscript including the literature search. GA is the senior author who described and used the technique. He also reviewed the article and suggested final changes before submission.
